# Evidence of a chimpanzee-sized ancestor of humans but a gibbon-sized ancestor of apes

**DOI:** 10.1038/s41467-017-00997-4

**Published:** 2017-10-12

**Authors:** Mark Grabowski, William L. Jungers

**Affiliations:** 1 Paleoanthropology, Senckenberg Centre for Human Evolution and Palaeoenvironment (HEP), Eberhard Karls University of Tübingen, Tübingen, Germany; 2 Division of Anthropology, American Museum of Natural History, New York City, NY 10024 USA; 3 Department of Biosciences, Centre for Ecological and Evolutionary Synthesis (CEES), University of Oslo, Oslo, 0316 Norway; 4 Department of Anthropology, Center for the Advanced Study of Human Paleobiology, The George Washington University, Washington, DC 20052 USA; 5 Association Vahatra, Antananarivo 101, BP 3972 Madagascar; 6 Department of Anatomical Sciences, Stony Brook University, Stony Brook, NY 11794 USA

## Abstract

Body mass directly affects how an animal relates to its environment and has a wide range of biological implications. However, little is known about the mass of the last common ancestor (LCA) of humans and chimpanzees, hominids (great apes and humans), or hominoids (all apes and humans), which is needed to evaluate numerous paleobiological hypotheses at and prior to the root of our lineage. Here we use phylogenetic comparative methods and data from primates including humans, fossil hominins, and a wide sample of fossil primates including Miocene apes from Africa, Europe, and Asia to test alternative hypotheses of body mass evolution. Our results suggest, contrary to previous suggestions, that the LCA of all hominoids lived in an environment that favored a gibbon-like size, but a series of selective regime shifts, possibly due to resource availability, led to a decrease and then increase in body mass in early hominins from a chimpanzee-sized LCA.

## Abstract (web summary)

The pattern of body size evolution in hominids can provide insight into historical human ecology. Here, Grabowski and Jungers use comparative phylogenetic analysis to reconstruct the likely size of the ancestor of humans and chimpanzees and the evolutionary history of selection on body size in primates.

## Introduction

Body mass impacts almost every aspect of an animal’s biology and ecology. Locomotion, behavior, diet, social organization, energy requirements, and a host of other vital biological and ecological characteristics are directly or indirectly tied to body mass. Thus, understanding the evolution of this trait is a necessary step in reconstructing the paleobiology of extinct fossil species. Though the timing, causes, and biological implications of the increase in body mass that took place during human evolution continue to inspire a wealth of research (e.g., refs. ^1–5^), the body mass of the last common ancestor (LCA) of chimpanzees and humans remains unexplored in any rigorous fashion. This omission is startling because numerous arguments over one of the most contested topics in hominin evolution—what were the selective regimes that led to the origins of bipedalism^6–9^ (but see ref. ^10^)—depend on inferences about body mass at and prior to the root of our lineage. Various classic models^9, 11–13^ proposed a body mass increase as a proximate factor in the evolution of suspensory adaptations and the transition from an arboreal to terrestrial hominid (great apes plus humans and our fossil ancestors) as larger sizes dictated a switch between locomotor modes, while models based around an arboreal quadruped ancestor (e.g., ref. ^14^) implicitly assumed a smaller-body mass in order to maintain balance and stability on deformable branches of different diameters^15^. Note that here we define body size as body mass^16^.

One important reason for this omission is the paucity of African fossil hominids during the period when the chimpanzee and human lineages are believed to have diverged, perhaps 4–6 Ma (million years ago)^17^ or earlier at 6–8 Ma^18–20^, with the notable exceptions of putative basal hominins *Orrorin tugenensis* (~6 Ma)^21^, *Sahelanthropus tchadensis* (6–7 Ma)^22^, *Ardipithecus kadabba* (5.5–6.4 Ma)^23^, and the later *Ardipithecus ramidus* (4.4 Ma)^7^. In addition, body sizes in the more well-sampled Miocene hominoid (all living and extant apes and humans) taxa (e.g., *Proconsul*) appear to be extremely variable (e.g., refs. ^24, 25^), and questions about how these species relate to one another and to crown hominoids (reviewed in ref. ^26^) further complicate the usefulness of these data. Issues with body mass variation in Miocene hominoids are evident in the description of *Ar. ramidus*, which argues that the chimpanzee-human and African hominid LCAs were likely to be “equal to or smaller than *Ar. ramidus*, possibly even substantially so”^7^—a range extending down from the fossil’s predicted body mass (~50 kg)^7^ and encompassing almost all primates from chimpanzees to diminutive monkeys. Another primary reason for this omission may be a consensus among many researchers that—reinforced by the wealth of molecular work showing humans and chimpanzees are sister taxa^27^—the mass of the chimpanzee–human LCA resembled common chimpanzees^28^ (~45 kg), and a chimpanzee-sized ancestor represents the LCA of African hominids (i.e., hominines)^10^, hominids^10^, and hominoids^29^. However, a few recent findings argue caution with acceptance of a chimpanzee-sized series of LCAs stretching back to before the divergence of hylobatids from other hominoids around 19.5 Ma. First, this hypothesis coincides with the assumption of an overall chimpanzee-like morphology for the chimpanzee–human and hominid LCA, a topic of much debate^6, 7, 30–33^, with some researchers suggesting that current fossil evidence and analyses point to a generalized monkey-like ancestor^34^. Chimpanzee-like postcranial morphology and body mass are not necessarily linked, although this is often implied by many models that suggest a chimpanzee-like LCA. Second, the description of *Pliobates cataloniae*, a small-bodied (4–5 kg) hominoid from the Miocene of Spain (11.6 Ma), argues for a gibbon-sized common ancestor of all crown hominoids, rather than an extant great ape-sized ancestor with hylobatids evolving as a dwarfed lineage^29^. Finally, a large-scale analysis of hominin body mass found earlier hominins were smaller-bodied than previously thought^3^ (Table 1, Supplementary Table 1), with no evidence for an orderly increase in body mass from *Australopithecus* to early (non-*erectus*) *Homo* to *Homo erectus* as has been suggested^35^. Average body mass for the well sampled *Australopithecus afarensis* was ~5 kg less than an average common chimpanzee, and many other well sampled later hominin taxa (*Australopithecus africanus*, possible *Paranthropus boisei*, *Paranthropus robustus*, *Homo habilis sensu stricto*) are ~5–10 kg below *Au. afarensis* (Table 1). While body mass predictions for the earliest undisputed hominin, *Australopithecus anamensis* (46.3 kg)^3^, and the earliest putative hominins *O. tugenensis* (35–50 kg)^36^, and the later *Ar. ramidus* (~50 kg) (but see refs. ^3, 7^), are all in the range of common chimpanzees, these estimates are based on single fossils^3^, and overall these findings argue that the pattern of body mass evolution in our own lineage may be more complicated than either stasis or a steady increase in body mass from a chimpanzee-like ancestor. Taken together, while a chimpanzee-sized LCA has been hypothesized as the phenotype from which all hominoid branches diverged, to the best of our knowledge this has not been tested in any quantitative, phylogenetically informed fashion. In addition, the data underlying these hypotheses appear to be problematic and further compounded by poor understanding of the Miocene fossil relationships, and likely heavily influenced by the view that chimpanzees provide fairly clear windows into our evolutionary past (see Supplementary Note 1 for taxonomic scheme used here).

**Table 1:** Estimated body mass averages, first appearance dates, sample sizes, and family designations for hominin and fossil taxa included in this analysis, along with comparative data for modern humans, *Pan troglodytes*, and *Pan paniscus*

Species	Average body mass (kg)	First appearance date (Ma)	Sample size	Family
*Orrorin tugenensis* ^*a*^	35.8/46.5	6.00	2	Hominidae
*Ar. ramidus* ^*a*^	32.1/50.8	4.40	1	Hominidae
*Au. anamensis*	46.30	4.17	1	Hominidae
*Au. afarensis*	39.10	3.77	12	Hominidae
*Au. africanus*	30.50	3.03	5	Hominidae
*Au. sediba*	26.70	1.95	2	Hominidae
*P. boisei* ^*b*^	35.30	2.30	8	Hominidae
*P. robustus*	30.10	2.00	4	Hominidae
*H. habilis*	32.60	2.33	2	Hominidae
*H. erectus*	51.00	1.90	7	Hominidae
*H. floresiensis*	27.50	0.10	1	Hominidae
*H. heidelbergensis*	69.10	0.61	5	Hominidae
*H. neanderthalensis*	75.40	0.13	14	Hominidae
*H. sapiens*	58.20	0.20	51^c^	Hominidae
*Proconsul africanus* ^d^	35.00	22.50	-	Proconsulidae^e^
*Proconsul major* ^d^	75.00	22.50	-	Proconsulidae^e^
*Ekembo heseloni* ^d^	15.00	18.50	-	Proconsulidae^e^
*Ekembo nyanzae* ^d^	35.00	18.50	-	Proconsulidae^e^
*Dryopithecus fontani*	43.60	11.80	1	Hominidae^e^
*Hispanopithecus laietanus*	32.00	9.60	2	Hominidae^e^
*Sivapithecus indicus*	30.50	12.70	6	Hominidae^e^
*Pliobates cataloniae*	4.50	11.60	1	Pliobatidae
*Pan troglodytes*	45.00	2.33	60	Hominidae
*Pan paniscus*	39.10	2.33	13	Hominidae

Full references and expanded information in Supplementary Table 1

^a^From ref. ^3^ based on chimpanzee training sample from ref. ^30^

^b^Based on “Possible *P. boisei*” following ref. ^3^

^c^Average of 51 worldwide populations following ref. ^2^

^d^Midpoint of range compiled in ref. ^68^

^e^Following ref. ^74^

To test alternative hypotheses on primate body mass evolution, we employ phylogenetic comparative methods^37–39^ on body mass data from a large sample of extant primates, a wide sample of fossil primates including Miocene fossil apes, and recently published data for fossil hominins^3^ to reconstruct the path of body mass evolution in individual branches of the primate phylogeny. Our fossil sample includes stem fossil apes *Proconsul africanus* (22.5 Ma) *Proconsul major* (22.5 Ma), *Ekembo* (*Proconsul*) *nyanzae* (20.0 Ma), *Ekembo* (*Proconsul*) *heseloni* (20.0 Ma), and *Pliobates cataloniae* (11.6 Ma), early hominids *Dryopithecus fontani* (11.8 Ma), *Hispanopithecus laietanus* (9.6 Ma), and *Sivapithecus indicus* (12.7 Ma), very early putative hominins *Orrorin tugenensis* (6.0 Ma) and *Ardipithecus ramidus* (4.4 Ma), and unequivocal hominins *Australopithecus anamensis* (4.17 Ma), *Australopithecus afarensis* (3.77 Ma), *Australopithecus africanus* (3.03 Ma), *Australopithecus sediba* (1.98 Ma), *Paranthropus boisei* (2.3 Ma), *Paranthropus robustus* (2.0 Ma), *Homo habilis* (2.33 Ma), *Homo erectus* (1.9 Ma), *Homo heidelbergensis* (0.609 Ma), *Homo neanderthalensis* (0.13 Ma), *Homo floresiensis* (0.1 Ma), and modern humans (0.195 Ma) (Table 1; Supplementary Table 1). Our approach here translates hypotheses on adaptation in selective regimes (also known as adaptive regimes or zones) into explicit evolutionary models, tests alternate models against comparative data (including fossils) using a maximum-likelihood model selection framework, and infers details of evolutionary processes such as estimating optimal body sizes for a given selective regime. Species in a selective regime share a common selective factor, and the underlying cause of differences in selective regimes may be differences in this factor related to environment, habitat, locomotion, etc. ^37^. When the selective regime changes, the fitness landscape of functional traits changes along with the optimal values for traits such as body mass, which leads to adaptation of traits towards this new optimum. Here, we test hypotheses of when shifts in selective regimes occurred along a phylogeny and estimate new optima (i.e., the optimal body mass of the new regime) that coincide with regime shifts by reconstructing the macroevolutionary adaptive landscape for primate and hominin body size. This approach provides a new and novel source of information on a trait that directly influences numerous hypotheses on the paleobiology of the human lineage.

First, we focus on body mass evolution within the hominoid clade, and show that the LCA of humans and chimpanzees, the earliest hominins, and the ancestor of African hominids lived in a selective regime that favored a modern chimpanzee-like body mass and was likely chimpanzee-sized. Further, the ancestor of all hominoids evolved in a regime that favored a gibbon-like body size, supporting recent fossil findings^40^. Second, we compare details of the evolutionary process within the hominoid clade, including fossil apes and hominins, to a large sample of extant and fossil primates and show that while most of primate evolution is characterized by evolution towards only two optimal body sizes, hominids are unique among primates in having a substantially greater number of adaptive optima due to distinct selective regimes across evolutionary time.

## Results

### Body mass evolution in hominoids including fossils

To reconstruct the adaptive landscape for body mass in primates including hominins and test hypotheses on when regime shifts occurred, we used the recently introduced “SURFACE” approach^38^ that fits a series of evolutionary hypotheses via Ornstein-Uhlenbeck or OU stabilizing selection models^37^ to phenotypic species data related via a phylogeny and retains the hypotheses that best fits the data (seen in the lowest corrected Akaike Information Criterion or AIC_c_ score). OU models permit the realization of Simpson’s^41^ adaptive zones by allowing for the placement of selective regimes along different branches of a phylogeny, where species in each regime evolve toward a distinct trait optimum—in this case the optimal body mass for a given regime. In the OU model, species have their own local adaptive optima, the position of which is influenced by numerous selective factors. Species within the same selective regime are also pulled toward a “primary” adaptive optima that is influenced by the selective factor or factors that define that regime (e.g., arboreality as a factor could define a selective regime with a smaller bodied optimal body size). Evolutionary constraints (due to ancestry, genetic correlations, functional constraints, and so on) reduce the rate of adaptation given a change in the selective regime, and can lead to species' trait values that are quite distant from the primary optimum of a given selective regime^37^. Here, “optima” or “adaptive optima” always refer to primary adaptive optima, not local adaptive optima. OU models differ from the more commonly used Brownian motion (BM)-based models of evolution as they model the evolutionary process as the combination a deterministic pull toward adaptive optima and fluctuations around this optimum that result from unmeasured forces (e.g., other selective factors, genetic drift). As such, OU models are consistent with current views on evolutionary change^39, 42, 43^. Importantly, this approach also identifies convergence in regimes—whether distantly related species found similar optima. Here, because the selective regimes are not assigned a priori, distantly related species in convergent regimes are not necessarily under the same set of selective factors, though they share the same estimated optima.

**Fig. 1:**
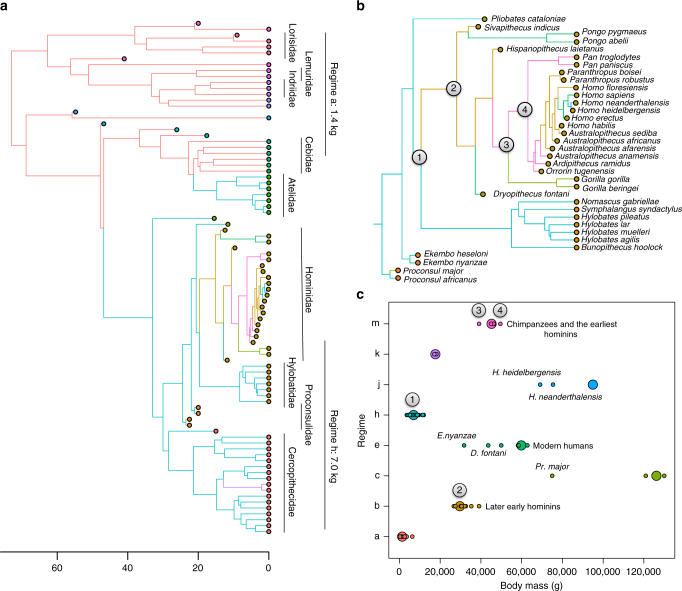
Time-calibrated phylogenetic tree with selective regimes and estimated body size optima. **a** Primate phylogenetic tree including fossils with tips color-coded to denote families, major families noted on the right. Phylogeny showing complete species names shown in Supplementary Fig. 1A Colors along branches showing best-supported selective regimes for body mass evolution including convergence and are consistent between **a**, **b** and **c**. Two major selective regimes for primates and optimal body size for each regime shown on far right correspond to Table 2; **b** focus on hominoids from **a** including fossil taxa. Marked nodes correspond to last common ancestors of all hominoids (1), hominids (2), African hominids (3), and chimpanzees and humans (4); **c** body mass averages (smaller circles) and inferred primary adaptive optima (larger circles) for species in each regime for primates including fossils corresponding to **a** and **b**. Numbered LCAs match nodes in **b**. Also noted is the adaptive optima of chimpanzees, the earliest hominins, later early hominins, and modern humans. Named taxa are outliers to their estimated optima

Our results show that the earliest putative hominins (*O. tugenensis*, *Ar. ramidus)*, and the early australopith *Au. anamensis* shared an selective regime with both species of *Pan* (Fig. 1a, b; Regime “m” in Table 2), and along with evidence that these fossil taxa were the mass of a chimpanzee (see above and Table 1), argue that the LCA of chimpanzees and humans was indeed chimpanzee-sized. This selective regime begins after the divergence of *Hispanopithecus* and includes the African hominid LCA—while geographically and temporally distinct, this result suggests that all of these species lived in an environment/or environments that favored the same optimal body size. While a selective regime favoring a slightly smaller body mass shared by *Sivapithecus* (with both species of *Pongo* sharing a derived larger body mass regime) and *Hispanopithecus* (Fig. 1a, b; Regime “b” in Table 2) was present after the divergence of hylobatids until after the LCA of hominids, the LCA of hominoids lived in a regime that was similar to extant hylobatids, stem fossil apes *Ekembo heseloni*, *Proconsul africanus*, and *Pliobates cataloniae*, and was plesiomorphic (primitive) and shared with a large number of Old World Monkey species and converged on by Atelidae (Fig. 1a, b; Regime “h” in Table 2). Focusing on hominins, a regime shift towards a smaller body mass (Fig. 1a, b; Regime “b” in Table 2) occurred with the arrival of *Au. afarensis* and persisted through all early hominins (*Au. africanus*, *Au. sediba, P. boisei, P. robustus*, *H. habilis*, *H*. *floresiensis*), before a regime shift to a larger optimal body mass near the origins of *H. erectus* shared with modern humans (Fig. 1a, b; Regime “e” in Table 2). There was also evidence that a few hominins and fossil apes converged on selective regimes shared with other hominoids—*Pr. africanus* converged on the body mass optima shared with both species of *Pan* and the earliest hominins, *Pr. major* with the large bodied regime of both *Gorilla* species, *E. nyanzae* and *D. fontani* with the larger bodied regime shared by both species of *Pongo*, which is also converged on by *H. erectus* and modern humans (Table 2).

**Table 2:** Selective regimes, estimated body mass optima, and groups assigned to each regime for primates including fossils with fossil families in parentheses

Regimes	Body mass optima (kg)	Assigned members	LCA
a	1.36	Cebidae, Cheirogaleidae, Daubentoniidae, Galagidae, Indriidae, Lemuridae, Lorisidae, Pithecidae, Tarsiidae, *Archicebus achilles* (Family: Archicebidae), *Eosimias sinensis* (Eosimiidae), *Karanisia clarki (*Galagidae), *Komba robustus* (Galagidae), *Carlocebus carmenensis* (Pitheciidae), *Nycticeboides simpsoni* (Lorisidae), *Branisella boliviana* (incertae sedis)	
b	29.68	*Hi. laietanus, S. indicus, Au. afarensis, Au. africanus, Au. sediba, H. floresiensis, H. habilis, P. boisei, P. robustus*	Hominid LCA
c	126.16	*Pr. major, G. beringei, G. gorilla*	
e	59.85	*E. nyanzae, D. fontani, H. erectus*, Modern humans*, Po. abelii, Po. pygmaeus*	
h	6.95	Atelidae, most Cercopithecidae, Hylobatidae, *Epipliopithecus vindobonensisi* (Pliopithecidae), *Ekembo heseloni* (Proconsulidae), *Proconsul africanus* (Proconsulidae), *Pliobates cataloniae* (Pliobatidae), *Victoriapithecus macinnesi* (Cercopithecidae)	Hominoid LCA
j	94.96	*H. heidelbergensis, H. neanderthalensis*	
k	17.65	*Papio anubis, Papio cynocephalus*	
m	45.23	*Ar. ramidus, Au. anamensis, O. tugenensis, Pa. paniscus, Pa. troglodytes, Pr. africanus*	African Hominid/Chimpanzee-Human LCA

LCA column denotes optima that include estimated body mass of the last common ancestor (LCA) of groups shown. Regimes match those in Fig. 1

**Fig. 2:**
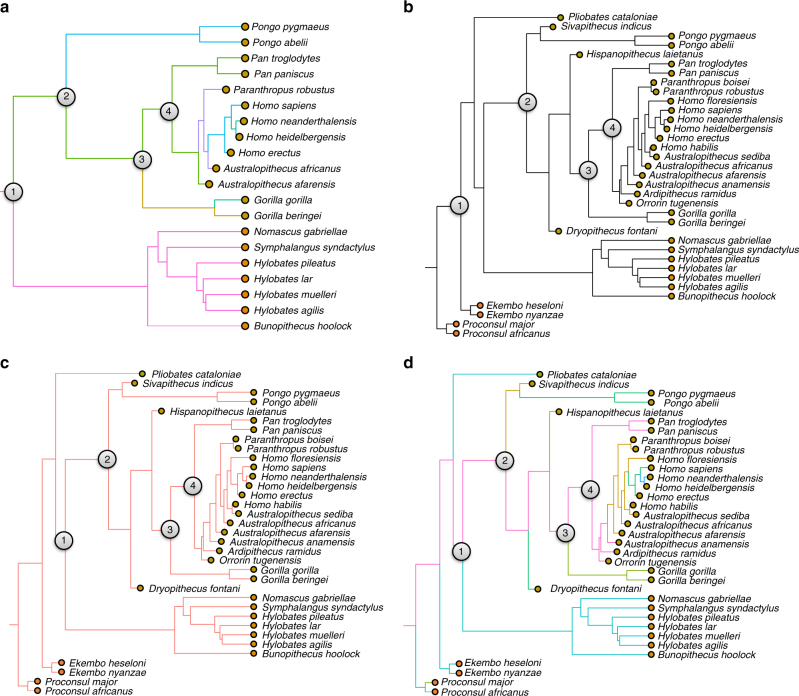
Alternative hypotheses for primates focused on hominoids. **a** Best-supported selective regimes with only well-sampled reliably attributed early hominins and without other fossil primates; complete data set with **b** Brownian motion; **c** single regime model (OU1); **d** chimpanzee*-*sized ancestor all hominoids. Colors reflect regime assignment within each figure and are not comparable between figures

To test how different estimates of body mass for *O. tugenensis* and *Ar. ramidus* ^7, 30^ affect our results, we reran our analysis using smaller body mass estimates that depend on the fossils sharing a modern human rather than a great ape pattern of scaling (Table 1). Our results (Supplementary Fig. 2A, B) show that a smaller body mass for these two taxa has no major impact on our overall findings—the LCA of chimpanzees and humans now shares a selective regime with the slightly smaller bodied pygmy chimpanzee (*Pa. paniscus*) as well as two of the earliest putative hominins (*O. tugenensis*, *Ar. ramidus*) but this regime now includes the slightly smaller *Au. afarensis* while *Au. anamensis* converges on the slightly larger-bodied regime that contains common chimpanzees. As the phylogenetic placement of *D. fontani* and other European and Asian hominids as closely related to African hominids is debated (reviewed in ref. ^26^), we tested how including these taxa affected our results by rerunning our analyses after removing these species. Overall results are consistent and robust (Supplementary Fig. 3A, B), with the only major change being that the larger-bodied regime that includes the *Pongo* lineage now includes both the hominid and the African hominid LCA, from which the smaller-bodied chimpanzee-like and larger bodied gorilla-like optima diverge. To test how inclusion of fossil taxa where average body mass estimates are based on only a few individuals (e.g., *n* = 1) affects our results, we reran our analyses using only those taxa where average body mass is based on more than two individuals (leading to the exclusion of *O. tugenensis*, *Ar. ramidus*, *Au. anamensis*, *Au. sediba*, *H. habilis*, and *H. floresiensis*) and are well attributed to particular taxa (leading to the exclusion of “probable” *P. boisei* ^3^). We also excluded the data from Miocene apes and other fossil primates because of uncertainties about their phylogenetic relatedness. Overall results (Fig. 2a; Supplementary Fig. 4) are extremely similar to the results using the smaller bodied estimates of *Ar. ramidus* and *O. tugenensis*—*Au. afarensis* shares a selective regime with *Pan* (here both *Pa. troglodytes* and *Pa. paniscus*) with a shift to a smaller-bodied regime early in australopiths (encompassing *Au. africanus* and *P. robustus*) and increase at the time of *H. erectus*, but now the *Pan*-sized selective regime extends back to a shift after the LCA of hominoids (Node “1”). Finally, to test how using species averages for extremely sexually dimorphic species affects our findings, we used only average female body mass for the extant primates and estimated female body mass averages for our well-sampled reliably attributed hominins^3^, leading to the removal of a number of hominin taxa (*O. tugenensis*, *Ar. ramidus*, *Au. anamensis*, *Au. sediba*, *P. boisei*, and *H. habilis*). Here (Supplementary Fig. 5), the LCA of chimpanzees and humans shares a selective regime with the smaller bodied pygmy chimpanzee (*Pa. paniscus*), which stretches back to shortly after the LCA of hominoids (Node “1”) and includes *Sivapithecus*, *Hispanopithecus*, *Pongo pygmaeus*, and *Au. afarensis*. It is important to note that in some lineages, female body masses may have evolved in a different manner than average body mass, as discussed below.

We tested the relative support of our results for each iteration of our data set compared to three other alternative evolutionary hypotheses (Fig. 2b–d) using relative AIC_c_ values including a hypothesis of evolution by genetic drift (a Brownian motion model of evolution), a model where adaptive evolution is toward one body mass optimum (a single-peak OU model), and a (multi-peak) model based on the SURFACE model where the LCA of all hominoids was chimpanzee-sized and the hylobatid lineage evolved by dwarfism. This last hypothesis assumes that the increase in body mass occurred prior to the divergence of the hylobatids and proconsuloids. Our results suggest that the adaptive landscape shown above (Fig. 1) and returned by SURFACE is the most likely model as seen in its smallest AIC_c_ values, regardless of the data set used (Table 3; Supplementary Table 2).

**Table 3:** AIC_c_ results and comparison between models for different evolutionary hypotheses including Brownian motion (BM), a single regime OU model (OU1), a chimpanzee-sized ancestor of all hominoids (Anc_*Pan*), and the best-supported model (Surface fit) for different subsets of body mass data

Data Set	Model	Surface AIC_c_	Change AIC_c_	*t*_1/2_
Complete data set (*N* = 87)	BM	1998.35	326.97	
	OU1	1968.73	297.36	
	Anc_*Pan*	1714.99	43.62	
	Surface fit	1671.37	0.00	0.88
Well sampled reliably attributed early hominins data set (*N* = 63)	BM	1418.45	225.58	
	OU1	1414.29	221.42	
	Anc_*Pan*	1315.41	122.54	
	Surface fit	1192.87	0.00	1.62
Extant primates only data set (*N* = 57)	BM	1265.85	193.73	
	OU1	1268.03	195.91	
	Anc_*Pan*	1080.731	8.61	
	Surface fit	1072.12	0.00	0.21

Also shown is half-life (*t*_1/2_) for each data set for the best-supported model

### Patterns of body mass evolution in primates

One main goal of the OU model of evolution is to infer details of evolutionary processes such as estimating adaptive optima for each selective regime and quantifying the rate of adaptation, measured by the phylogenetic half-life (*t*_1/2_)—the average time it takes for the trait to evolve half of the distance from its ancestral state to the new optima after a regime shift^37^. Half-lives provide an estimate of the time it takes before adaptation to the new selective regime is expected to be more influential than constraints from the ancestral regime, thus providing a metric quantifying the effects of phylogenetic inertia (resistance or slowness in adaptation to the optima). Combined with half-lives greater than zero, divergence of individual species means from their optimum can be an indication of constraints on adaptation from any source (e.g., genetic, selective, and so on). Our best-fit model shows that all non-hominid (and non-proconsuloid) primates are evolving towards only two body mass optima (regime “a” and regime “h” Fig. 1a)—with some groups converging on these optima even when separated by deep time (e.g., the Atelidae with Hylobatidae and the majority of Cercopithecidae, separated by about 43 Ma^44^). All species within these regimes appear relatively close to their optima—shown in the distance of the smaller circles (species means) from the larger circles (body mass optima) in Fig. 1c. Included extant strepsirrhine species from the families Cheirogaleidae, Daubentoniidae, Galagidae, Indriidae, Lemuridae, Lorisidae, and species of the New World Monkey group Cebidae, as well as extant taxa *Aotus trivirgatus*, *Tarsius bancanus*, and *Cacajao calvus* and fossil taxa *Archicebus achilles* (Family: Archicebidae), *Karanisia clarki* (Galagidae), *Komba robustus* (Galagidae), *Carlocebus carmenensis* (Pitheciidae), *Nycticeboides simpsoni* (Lorisidae), *Branisella boliviana* (incertae sedis), are all evolving toward an optimal body mass of around 1.4 kg (Regime “a” in Fig 1a, c; Table 2); extant species from the families Atelidae, Hylobatidae, and almost all Cercopithecidae, as well as fossil taxa *Victoriapithecus macinnesi* (Cercopithecidae), *Epipliopithecus vindobonensi* (Pliopithecidae), *Ekembo heseloni* (Proconsulidae), *Proconsul africanus* (Proconsulidae), and *Pliobates cataloniae* (Pliobatidae) are evolving toward an optimal mass of around 7.0 kg (Regime “h” in Fig 1a, c; Table 2). Undoubtedly, inclusion of large-bodied extinct species for some clades (e.g., subfossil “giant” lemurs and huge Pleistocene atelids) would impact some of these results, but including these groups would likely merely add side branches where body mass independently increased (such as seen in the *Gorilla* lineage) and have no effect on our overall results. Hominids and proconsuloids are evolving to five unique optima out of the eight estimated optima for primates (Fig. 1; Table 2) despite the relatively small number of species in this clade. Hominids also appear to show the greatest difference between species averages and the estimated optima, which, combined with half-lives around 1 Ma (see below), suggest body mass evolution is constrained in our clade and/or not enough time has passed for lineages to adapt to their new optima after a regime shift (Fig 1c). The largest differences between the estimated adaptive optima for a given regime and the species placed within that regime are *E. nyanzae* evolving toward the larger body mass optimum shared with modern humans (Regime “e” in Fig. 1c, Table 2), *Pr. major* evolving toward the regime shared by both species of *Gorilla* with an optimal value of 126 kg (Regime “c” in Fig. 1c; Table 2), and *H. heidelbergensis* and *H. neanderthalensis* (Regime “j” in Fig. 1c; Table 2), evolving towards a substantially larger body mass optimum of 95 kg. Extreme optima estimates could mean that evolution in a particular lineage is not well modeled by the current OU process, which assumes a constant rate of adaptation and constant magnitude of stochastic fluctuations (e.g., the lineage could be evolving via a Brownian-motion process), or the lineage was indeed experiencing directional selection to a distant optimum^45^. While an optimal body mass for either hominin species and the larger *Proconsul* taxon slightly below or approaching that of gorillas might seem unlikely, optima are the average trait values species within a regime would reach given enough time and free of constraints^37^. These results suggest that these large-bodied hominoids would have eventually evolved even larger body masses, and overall patterns are repeated using the data set with the smaller-bodied estimates of *Orrorin* and *Ardipithecus* (Supplementary Fig. 2). Importantly, species means fall extremely close to the estimated optima for the chimpanzee–human LCA (Node “4” in Fig. 1 and Regime “m” in Fig. 1c) and hominoid LCA (Node “1”, Regime “h”), providing good evidence that the body mass of the LCA in these regimes would be quite similar to the other species in the regime and close to the estimated optima. The half-life for all data sets of species averages including fossils is between 0.82 and 1.62 Ma (Table 3; Supplementary Table 2), meaning that it takes around a million years for the average primate to evolve half-way toward a new body mass optimum, which is extremely rapid evolution with only some influence from past history. Notably, when using only average female body mass for the extant primates and well-sampled female body mass averages for hominins from above, the half-life increases to 6.52 Ma (Supplementary Table 2). Relatively long half-lives could suggest that female body mass may be more constrained than average species body mass, likely because of a closer link between females and ecology^46–49^. Another possibility is that female body mass evolution on individual branches of the phylogenetic tree adapted at different rates than the majority of other lineages (see above and ref. ^45^). Support for this latter contention is suggested when comparing the estimated adaptive optima to the female averages, where some optima are quite distant from the taxa within their selective regime (Supplementary Fig. 5). It is of note that the AICc score of the current best fit SURFACE model is still substantially better (a difference of more than 200 units) than a Brownian-motion-based model or a model with only one adaptive peak (Supplementary Table 2), suggesting that neither other evolutionary model is more appropriate for modeling female body mass evolution.

To test how including fossil data affects our results, we reran the analysis with a phylogeny and data from only extant species (Supplementary Fig. 6). Though there are now only seven hominid species, this group again shows high variation in the number of body mass regimes, evolving towards four optima, or more than half of the total number of regimes for primates. One important change is that the LCA for hominids and African hominids as well as chimpanzees and humans now shares a selective regime with *Po. pygmaeus*, a regime that appears constant at nodes within the phylogeny up to both *Po. pygmaeus* and modern humans (Nodes “2”–“4”). Notably, the LCA of all hominoids (Node “1”) remains in a regime shared with hylobatids and a large number of other primates, suggesting that this body mass was optimal at this time period, though this could be driven by the presence of the deeply rooted hylobatids. Another important change is the half-life is now 0.21 Ma (Table 3), a significant decrease from previous runs and suggests that the addition of fossil data could provide a more accurate estimate of evolutionary parameters than analyses based purely on extant data (see ref. ^50^ for more on this point). Additionally, comparing exceedingly long half-lives of the extant females-only data set to the extant species averages data set (Supplementary Fig. 7), 10.13 vs. 0.21 Ma (Supplementary Table 2 vs. Table 3), provides further support that average female primate body mass may be evolving in a substantially different fashion than species averages.

## Discussion

The results of our novel comparative phylogenetic analysis of body mass evolution in primates have large consequences for the paleobiology of hominoid and hominin origins. First, our results suggest that the LCA of chimpanzees and humans lived in an environment that favored a body mass similar to modern chimpanzees (either *Pa. troglodytes*, *Pa. paniscus*, or both depending on the data set used), and this optimal body mass was shared with the earliest hominins. Consistent with fossil evidence of large body sizes, our results support earlier suggestions^9, 10, 28, 29^ that this LCA had a body mass close to that of modern chimpanzees. It should be noted that this regime persisted in the earliest hominins until shifting to a smaller-bodied regime near or following (depending on the data set used here) the origins of *Au. afarensis* at 3.77 M. While this reduction in the optimal average body mass could be due to a reduction in female body mass resulting from differential effects of ecological stresses^47^—such as caused by climate variability at Hadar 3.4–2.9 million years ago^51^—recent findings^3^ suggest that later early hominins from South Africa (*Au. africanus*, *P. robustus*) were smaller bodied both on average than earlier *Au. afarensis*, and purported males may have had a slightly larger decrease in body mass than females (~4%). Thus, if an increase in sexual dimorphism in *Au. afarensis* was the result of ecological stresses affecting females to a greater extent than males (e.g., refs. ^47–49^), it appears that these stresses affected the sexes in a more similar manner in later australopiths. In fact, the estimated optimal body mass for the later smaller-bodied early hominin regime was slightly below 30 kg (Table 2), which is about 10 kg smaller than the earlier *Au. afarensis*, and later early hominins (starting with the 30.5 kg *Au. africanus* at 3.03 Ma), including the small-bodied *H. habilis*, appear to be evolving towards this new smaller optimal body mass. The origin of *Au. africanus* coincides with the shift towards more open environments after 3 Ma. in South African sites^52^, as well as apparently increased greater climatic variability in East Africa^35^, which likely imposed new selective pressures on early hominins and led to a regime shift at this time. The regime shift to larger optimal body sizes near the origins of *H. erectus* (Fig. 1b, c) as well as larger body mass in this taxon (Table 1) could signify the combination of environmental changes to more favorable conditions or behavioral differences leading to shifts in the ability to use available resources (e.g., a greater reliance on high-quality sources such as meat^53^). Of course, this sequence of body mass evolution (and the results of this analysis) depends on the relationships among taxa, but at the very least there appears to be a substantial decrease in both the species average as well as average male and female body mass for hominins between 3.0 and 2.0 Ma with the extinction of *Au. afarensis* ^3^. It is also suggestive that the optimal body mass for the regime that contains *H. heidelbergensis* and *H. neanderthalensis* is slightly below the average mass of *G. gorilla* and *G. beringei* in almost all iterations here, and these hominins may in fact be evolving toward a selective regime that favored increasingly large body sizes due to factors such as colder climates^54, 55^ or hunting larger-bodied prey^56^. No doubt hominin body mass was constrained and influenced by a wealth of factors, such as sexual selection, food availability and other ecological influences, tool-use, and physiological constraints that are not tested in the current model. Taking a step back, it is also notable that we found primate female body mass adapting to new optima at a substantially slower rate than species data, seen in much larger half-life values (Supplementary Table 2), which argues that sexual dimorphism in body mass is the result of males and females responding to selection pressures on different time scales and/or selection pressures come from different sources. One possible reason that may be partially supported our findings for hominins is females appear to be more heavily influenced by the environment than males because female reproductive success depends on acquiring energetic resources for birth and lactation^48, 49, 57, 58^. Thus, one would expect the rate of adaptation for females to follow broad shifts in ecology—i.e., an adaptive landscape that is dominated by peaks defined by ecological factors—that likely occur on a much slower time scale than competing selective pressures on males (e.g., intraspecific sexual selection, along with ecological factors).

Second, our results indicate that the LCA of all hominoids shared a selective regime with hylobatids and was likely the mass of a modern gibbon, arguing against the view that hylobatids are a dwarf lineage from a great ape-sized ancestor of all hominoids (e.g., ref. ^29^). Larger mass apparently did not evolve until after the divergence of hylobatids, with two regime shifts to increasingly larger body mass optima prior to the LCA of hominids. While we include stem ape *Pliobates cataloniae* in our main analyses, our findings without Miocene ape taxa (Supplementary Fig. 3) independently support Alba et al.’s^40^ recent claim, and earlier suggestions^8, 9^ of a gibbon-sized ancestor of hominoids based on the type specimen of *Pliobates cataloniae*. We also note that this body mass regime is also shared by the majority of Old World Monkeys and by the distantly related New World Monkey family Atelidae, and may be the plesiomorphic (ancestral) condition for catarrhines. While it was suggested that that suspensory behavior in hominoids evolved as a necessary locomotor shift coinciding with increasing body mass^15^, a gibbon-sized ancestor of all apes argues against this hypothesis—it could be that antipronogrady first evolved in a gibbon-sized early ape, further adapting in the lineage that led to hylobatids. An adaptive shift favoring a larger body mass could have led some early hominoids—already adapted to a rudimentary form of suspensory locomotion—to adapt their morphology and behavior to deal with this change, leading to some of the differences between great ape and gibbon locomotor behavior^59^. In this model, there is no need for the independent acquisition of suspensory behavior among the hominoid lineages—the series of morphological changes that allow for suspensory behavior evolved once and the combination of continued use and possibly phylogenetic inertia (resistance or slowness in adaptation) in these characters led to their persistence while body mass appears to be extremely evolvable in this clade. Taking a step back, suspensory behavior and increased body mass have been argued to be hominoid adaptations to a foraging strategy allowing them to compete with increasingly numerous old world monkeys since the Middle Miocene (reviewed in ref. ^60^). Our results suggest that these two adaptations occurred independently of each other and could have been part of an arms race with monkeys for fruit resources—suspensory behavior to access ripe fruit on compliant branches at the edges of foliage evolved first, followed by larger body sizes when direct physical competition was required. Sexual selection in hominids likely further increased optimal average body sizes. We also note that although hominin and hominoid evolution is the focus of this analysis, our complete results suggest that the basal euprimate lived in a selective regime that favored an optimal body mass between 1.4 and 1.6 kg (e.g., Fig. 1). Though this estimate is close to previous suggestions^61^, it is far above an analysis that included body mass estimates for early primate fossils (~55 g)^19^ (but see ref. ^62^).

Finally, our results provide evidence of a complex and changing adaptive landscape in the hominin and hominid clades—while almost all other primates are evolving toward two body mass optima in our sample (e.g., 1.4 and 7.0 kg; Fig. 1a), hominids (including proconsuloids) had a substantially greater number of adaptive optima due to distinct regime shifts than any other group (Fig. 1c). While these results are preliminary, they suggest that most of primate evolution has taken place within a small number of ecological niches—one small-bodied regime principally based around arboreal quadrupedalism and leaping, one larger bodied regime, members of which evolved toward suspensory behavior (Hylobatidae and Atelidae) and continued arboreal quadrupedalism and leaping (most Cercopithecidae). Larger-bodied species adapted to terrestrial locomotion—*Pa. cynocephalus* and *Pa. anubis* here, are near their own body mass optima (Regime “k”—17.7 kg; Table 2). Within each group is variation in locomotor behavior, as well as diet, social structure, and so on—differing local selective pressures that likely led to the variation around the optimal body mass within a given regime. Together, the greater number and greater complexity of body mass optima for hominids and hominins supports the hypothesis that dramatic and uncommon shifts in the adaptive landscape drove human evolution.

## Methods

### Body mass averages

Extant primate body mass averages were taken from Isler et al.^63^, which focused on wild-caught primates, supplemented by data for modern humans from Ruff et al.^2^. Early fossil hominin body mass species averages were taken from Grabowski et al.^3^, supplemented by previously published data on hominins and Miocene fossil apes (Table 1; see Supplementary Table 1 for complete references). Body mass of *Ar. ramidus* is uncertain^3, 7^ based on whether this species scaled more like a modern human or chimpanzee. For *ARA-VP-6/500* we used the modern human-based estimate of 32.1 kg^3^ and the chimpanzee-based estimate 50.8 kg^30^. We used a similar approach for the body mass of *O. tugenensis*—the modern human-based estimate of 35.8 kg was taken from Grabowski et al.^3^ and we calculated a chimpanzee-based estimate using the supero-inferior femoral head diameter (FHD) of 33.2 mm for BAR 1002’00 and the regression of FHD on body mass for 28 chimpanzees from Almécija et al.^30^.

### Phylogeny

We used a composite phylogeny based on the dated consensus tree from version 3 of 10Ktrees^64^, the latest phylogeny for fossil hominins from Dembo et al.^65^, and the addition of the stem fossil ape species *Pr. africanus* (22.5 Ma), *Pr. major* (22.5 Ma), *E. nyanzae* (20.0 Ma), *E. heseloni* (20.0 Ma)^66^, and *P. cataloniae* (11.6 Ma), European fossil hominids *D. fontani* (11.8 Ma), and *Hi. laietanus* (9.6 Ma), and Asian fossil hominid *S. indicus* (12.7 Ma) based on their first appearance and proposed phylogenetic relationships. Both species of *Ekembo* were formerly placed in *Proconsul* but united into their own genus based on morphology^66^. Here *Ekembo* and *Proconsul* form two clades^66^ and diverge separately from the main trunk prior to the divergence of hylobatids. *Proconsul* (and by extension *Ekembo*) is accepted as a stem hominoid by the majority of researchers^67^ although some argue that it is a stem catarrhine^68^. We placed all five early Miocene apes as originating shortly before the divergence of hylobatids, later Miocene great ape *Dryopithecus* originating before the *Gorilla* lineage, followed by *Hispanopithecus*, with *Sivapithecus* on the branch that led to *Pongo*. For these fossil species, ghost lineages of 1 Ma were added to the published age of the fossil as a criterion for standardization. We also updated the divergence date for *H. floresiensis* based on recent findings^69^. We added a wide array of non-hominoid fossil primates (Table 1) to our phylogeny based on their first appearance dates and proposed relationships from a time scaled phylogeny of living and extinct primates^19^. As most of these non-hominoid fossil taxa averages are based on rare single individuals, we assumed that average body size and female body size were the same for our females-only analyses above. See Supplementary Fig. 1 for complete phylogeny with species names, Supplementary Data 1 for phylogeny with branch lengths. To focus on hominoid evolution within a larger primate context, as well as decrease computation time and redundancy in our phylogeny, we reduced the number of species in our extant data set from 176 in Isler et al.^63^ to 87 for our main analysis. To do this, we first took the subset of species averages from Isler et al.^63^ that are based on at least one male and one female individual and therefore are the more reliable estimates of species means^70^—totaling 161 species. After matching these species with those in our phylogeny (see below) we used a routine where given sister species of roughly similar mass (sister species where the second sister species was > 80% the mass of the first), the second species in each comparison was dropped from the analysis, leaving 111 non-hominid species. We then combined in our fossil and extant hominid data set (Table 1; Supplementary Table 1). Finally, the tree was pruned to 87 species for our complete data set by removing random non-hominid extant branches.

### Comparative approach

Our phylogenetic comparative approach is based around the model of adaptive evolution introduced by Hansen^37^ and implemented in the R package “SURFACE” (“SURFACE Uses Regime with Akaike Information Criterion (AIC) to model Convergent Evolution”)^38^, fitted in R 3.2.3^71^. While a purely neutral model of evolution—Brownian motion—underlies most comparative approaches for quantitative characters^72^, the expected value of a trait remains equal to its ancestral value, and thus this model does not make allowance for the tendency of traits to adapt in response to selection. In Hansen’s^37^ adaptive model, trait change is modeled according to an Ornstein-Uhlenbeck (OU) process, where the change in a quantitative trait *X* is the sum of a deterministic part (interpreted as the force of selection acting on the trait) and a stochastic part (interpreted fluctuations in the local fitness optima due to unmeasured factors). This can be expressed as the stochastic differential equation^39^:

1 $${\rm{d}}X\left( t \right) = \alpha [ {\theta - X\left( t \right)} ]{\rm{d}}t + \sigma {\rm{d}}B\left( t \right)$$

where d*X*(*t*) is the change in *X*, in a small time interval, d*t*, d*B*(*t*) is a white-noise process (i.e., independent, normally distributed random changes with mean zero and unit variance), and *σ* measures the intensity of random fluctuations in the evolutionary process. The term *θ*−*X*(*t*) quantifies the distance of the current trait value of *X* from the optimum for a given regime, *θ*, and is proportional to the force of selection, increasing the farther the trait value is from the optimum. The parameter *α* quantifies the rate of adaptation; when *α* = 0 the deterministic part of the equation drops out and the model becomes akin to Brownian motion. *α* can be expressed in terms that allow comparison to the overall length of the phylogeny as a phylogenetic half-life, *t*_1/2_ = ln 2/*α*, the time it takes for a maladapted species to evolve half the distance to the optimum on average. A short half-life would mean rapid adaptation towards the optimum; a long half-life would mean that adaptation is slow and that species tend to be poorly adapted relative to their optimum. A half-life of infinity means there is no tendency to evolve toward the optimum, and evolution is akin to Brownian motion. The R package “OUCH” (“Ornstein-Uhlenbeck models for phylogenetic comparative hypotheses”)^39^ was the first to allow for testing of alternative hypotheses on adaption in selective regimes by translating these hypotheses into explicit evolutionary models and testing alternate models against comparative data using a maximum-likelihood (ML) model selection framework. SURFACE^38^ builds on the approach and functions in OUCH by locating selective regimes without a priori hypotheses, where regime fitting is dictated by the phenotypic data and adaptive shifts are placed on the branches of a phylogenetic tree that most improve the corrected Akaike Information Criterion (AIC_c_) score. SURFACE begins its stepwise model selection routine an OU model with one adaptive peak (OU1 in Fig. 2c), and fits increasingly complex models specifying an increasing number of peaks, retaining new peak shifts that most improve the model fit until no improvement is possible. Importantly, this approach also identifies convergence in regimes—whether distantly related species found similar optima—by testing whether further improvement in AIC_c_ score is possible by allowing different lineages to evolve toward the same adaptive peak. Here, because the selective regimes are not assigned a priori, distantly related species in convergent regimes are not necessarily under the same set of selective factors, though they share the same estimated optima. While SURFACE performs best on multiple traits^38^, with single traits such as used here there is only a relatively slight decline in the accuracy of its grouping of tip species into regimes, with a greater effect on the ability to detect deep regime shifts along the phylogenetic tree. Here we are most interested in the selective regimes in which the taxa at the tips evolved, and inferring the regimes of their common ancestor based on these results, and thus using a single trait is justified for our overall evolutionary question. In addition, our analysis includes fossil primates separated by millions of years and across a broad number of different clades, which has been shown to substantially increase the precision of estimated optima and placement of selective shifts on the phylogeny (see also refs. ^50, 73^).

### Data availability

All fossil and modern human data analyzed in this study are included in this published article. All extant data analyzed in this study were previously published in Isler et al.^63^ but can be made available upon a reasonable request.

## Electronic supplementary material

Supplementary Information

Description of Additional Supplementary Files

Supplementary Data
