# Supplementary material for: Evidence of a chimpanzee-sized ancestor of humans but a gibbon-sized ancestor of apes
Source: Nat Commun. 2017 Oct 12;8:880. doi: 10.1038/s41467-017-00997-4 (PMC5638852; doi:10.1038/s41467-017-00997-4)
Supplement: Supplementary file 1 — Supplementary Information [file 41467_2017_997_MOESM1_ESM.pdf]

Supplementary Note 1:
The taxonomic scheme in this manuscript follows:

Superfamily Hominoidea (hominoids)
- Family Proconsulidae
  - Genus *Proconsul*
  - Genus *Ekembo*
- Family Pliobatidae
  - Genus *Pliobates*
- Family Hylobatidae (hylobatids)
  - Genus *Hylobates*
- Family Hominidae (hominids)
  - Subfamily Dryopithecinae
    - Genus *Dryopithecus*
    - Genus *Hispanopithecus*
  - Subfamily Ponginae (pongines)
    - Genus *Pongo*
    - Genus *Sivapithecus*
  - Subfamily Homininae (hominines)
    - Tribe Gorillini (gorillins)
      - Genus *Gorilla*
    - Tribe Panini (panins)
      - Genus *Pan*
    - Tribe Hominini (hominins)
      - Genus *Orrorin**
      - Genus *Sahelanthropus**
      - Genus *Ardipithecus**
      - Genus *Australopithecus*
      - Genus *Paranthropus*
      - Genus *Homo*

* putative basal hominin

Supplementary Table 1: Estimated body mass averages, first appearance dates, sample sizes, and references for fossil taxa included in this analysis, along with comparative data for modern humans, *Pan troglodytes*, and *Pan paniscus*.

**Species**	**Average Body Mass (kg)**	**First Appearance Date (Ma)**	**Sample size**	**Family**	**Reference for Mass**	**Reference for Age**
Orrorin tugenensis[α]	35.8/46.5	6.00	2	Hominidae	Grabowski et al. 2015[1] / Almécija et al. 2015[2]	Almecija et al. 2013[16]
Ar. ramidus[α]	32.1/50.8	4.40	1	Hominidae	Grabowski et al. 2015[1] / Almécija et al. 2015[2]	White et al. 2009[18]
Au. anamensis	46.30	4.17	1	Hominidae	Grabowski et al. 2015[1]	Leakey et al. 1998[18]
Au. afarensis	39.10	3.77	12	Hominidae	Grabowski et al. 2015[1]	Leakey et al. 1976[19]
Au. africanus	30.50	3.03	5	Hominidae	Grabowski et al. 2015[1]	Herries et al. 2013[20]
Au. sediba	26.70	1.95	2	Hominidae	Grabowski et al. 2015[1]	Pickering et al. 2011[21]
P. boisei[β]	35.30	2.30	8	Hominidae	Grabowski et al. 2015[1]	Feibel et al. 1989[22]
P. robustus	30.10	2.00	4	Hominidae	Grabowski et al. 2015[1]	Herries et al. 2009[23]
H. habilis	32.60	2.33	2	Hominidae	Grabowski et al. 2015[1]	Kimbel et al. 1997[24]
H. erectus	51.00	1.90	7	Hominidae	Grabowski et al. 2015[1]	Kimbel and Villmoare, 2016[25]
H. floresiensis	27.50	0.10	1	Hominidae	Grabowski et al. 2015[1]	Sutikna et al. 2016[26]
H. heidelbergensis	69.10	0.61	5	Hominidae	Arsuaga et al. 2015[3]	Wagner et al. 2010[27]
H. neanderthalensis	75.40	0.13	14	Hominidae	Arsuaga et al. 2015[3]	Rink et al. 1995[28]
Modern Humans	58.20	0.20	51[ε]	Hominidae	Ruff et al. 1997[4]	McDougall et al. 1995[29]
Proconsul africanus[δ]	35.00	22.50	-	Proconsulidae[ϕ]	Harrison 2010[5]	McNulty et al. 2015[30]
Proconsul major[δ]	75.00	22.50	-	Proconsulidae[ϕ]	Harrison 2010[5]	McNulty et al. 2015[30]
Ekembo heseloni[δ]	15.00	18.50	-	Proconsulidae[ϕ]	Harrison 2010[5]	McNulty et al. 2015[30]
Ekembo nyanzae[δ]	35.00	18.50	-	Proconsulidae[v]	Harrison 2010[5]	McNulty et al. 2015[30]
Dryopithecus fontani	43.60	11.80	1	Hominidae[ϕ]	Moya-Sola et al. 2009[6]	Moya-Sola et al. 2009[6]
Hispanopithecus laietanus	32.00	9.60	2	Hominidae[ϕ]	Alba et al. 2012[7]	Alba et al. 2012[7]
Sivapithecus indicus	30.50	12.70	6	Hominidae[ϕ]	Morgan et al. 2015[8]	Morgan et al. 2015[8]
Pliobates cataloniae	4.50	11.60	1	Pliobatidae	Alba et al. 2015[9]	Alba et al. 2015[9]

Komba robustus	0.30	20.00	-	Galagidae	Steiper and Seiffert, 2012[10]	Harrison, 2010[5]
Nycticeboides simpsoni	0.27	9.00	-	Lorisidae	Steiper and Seiffert, 2012[10]	Harrison, 2010[5]
Epipliopithecus vindobonensi	11.50	15.50	1	Pliopithecidae	Alba et al. 2015[9]	Begun, 2002[31]
Carlocebus carmenensis	3.37	17.50	-	Pitheciidae	Steiper and Seiffert, 2012[10]	Fleagle, 2013[32]
Branisella boliviana	0.48	26.00	-	incertae sedis	Steiper and Seiffert, 2012[10]	Takai et al. 2000[33]
Karanisia clarki	0.27	41.00	1	Galagidae	Seiffert et al. 2003[11]	Harrison, 2010[5]
Victoriapithecus macinnesi	3.75[3]	15.00	-	Cercopithecidae	Harrison, 1989[12]	Gonzales et al. 2015[34]
Archicebus achilles	0.03	55.80	1	Archicebidae	Ni et al. 2013[13]	Ni et al. 2013[13]
Eosimias sinensis	0.14	45.00	1	Eosimiidae	Egi et al. 2004[14]	Beard and Wang. 2004[35]
Pan troglodytes	45.00	2.33	60	Hominidae	Smith and Jungers 1997[15]	10K Trees[36]
Pan paniscus	39.10	2.33	13	Hominidae	Smith and Jungers 1997[15]	10K Trees[36]

α From [1]/Based on chimpanzee training sample from[2]

β Based on "Possible *P. boisei*" following[1]

δ Midpoint of range compiled in[5]

ε Average of 51 worldwide populations

ϕ Following[6]

Supplementary Table 2: AIC_c results and comparison between models for different evolutionary hypotheses including Brownian-motion (BM), a single regime OU model (OU1), a chimpanzee sized ancestor of all hominoids (Anc_*Pan*), and the best supported model (Surface fit) for different subsets of body mass data. Also shown is half-life ($t_{1/2}$) for each data set for the best-supported model.

Data Set	Evolutionary Model	AIC_c	Change AIC_c	$t_{1/2}$
**Complete Data Set with Smaller *Ardipithecus* and Orrorin Estimates (n=87)**	**BM**	1999.38	330.12	
	**OU1**	1969.42	300.16	
	**Anc_*Pan***	1680.79	11.52	
	**Surface fit**	1669.27	0.00	0.82
**Without Miocene Apes (n=79*)***	**BM**	1759.10	263.06	
	**OU1**	1753.11	257.07	
	**Anc_*Pan***	1500.62	4.59	
	**Surface fit**	1496.04	0.00	0.85
**Female Data Only - Well Sampled Early Hominins with other Fossil Primates (n=78)**	**BM**	1711.95	258.52	
	**OU1**	1700.86	247.43	
	**Anc_*Pan***	1502.52	49.10	
	**Surface fit**	1453.42	0.00	6.52
**Female Data Only - Extant Primates (n=*57)***	**BM**	1233.95	177.33	
	**OU1**	1232.95	176.33	
	**Anc_*Pan***	1068.79	12.18	
	**Surface fit**	1056.62	0.00	10.10

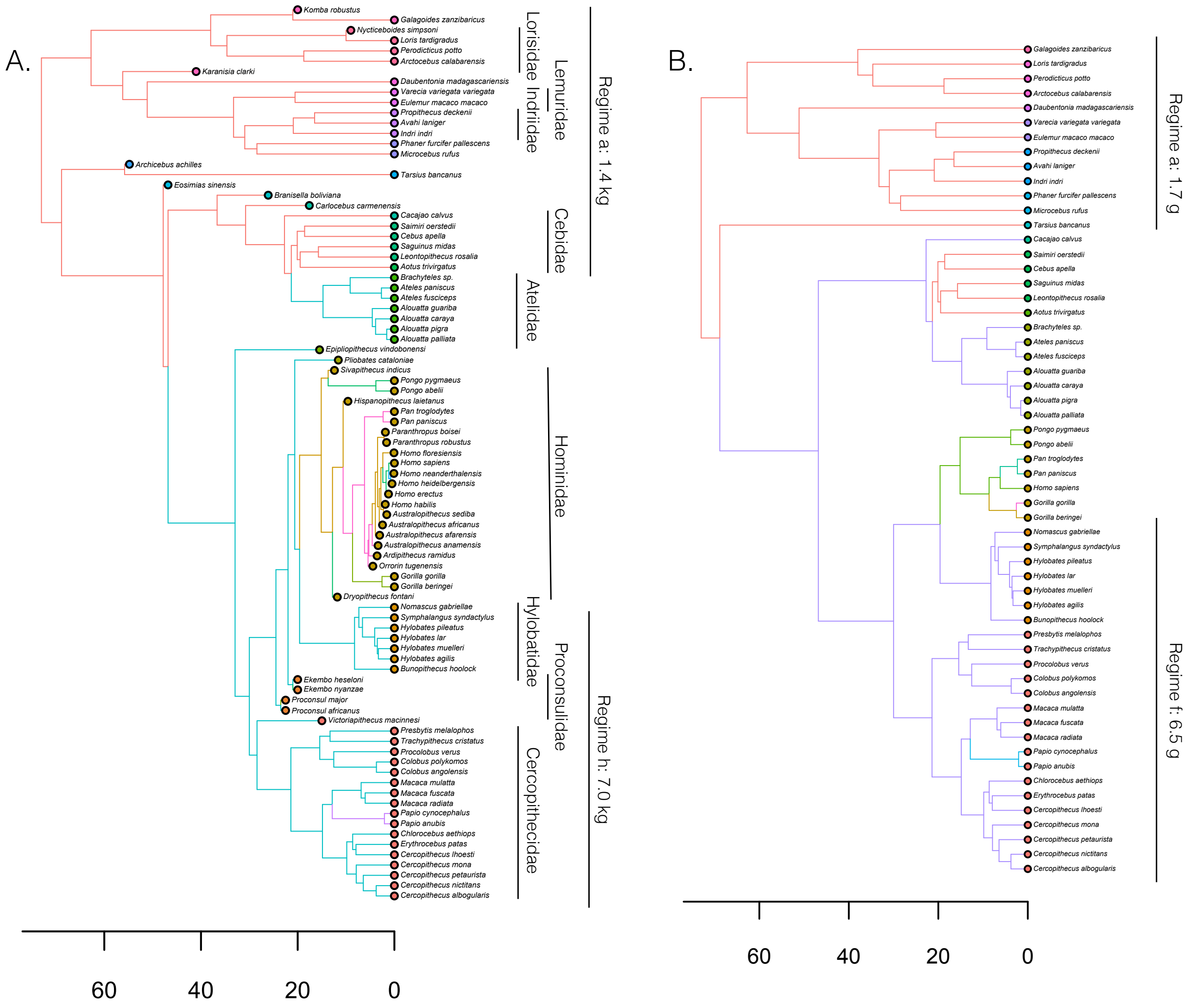

Supplementary Figure 1: A) Same as Figure 1:A except with species names; B) Same as Figure 1:A except using only extant primate data.

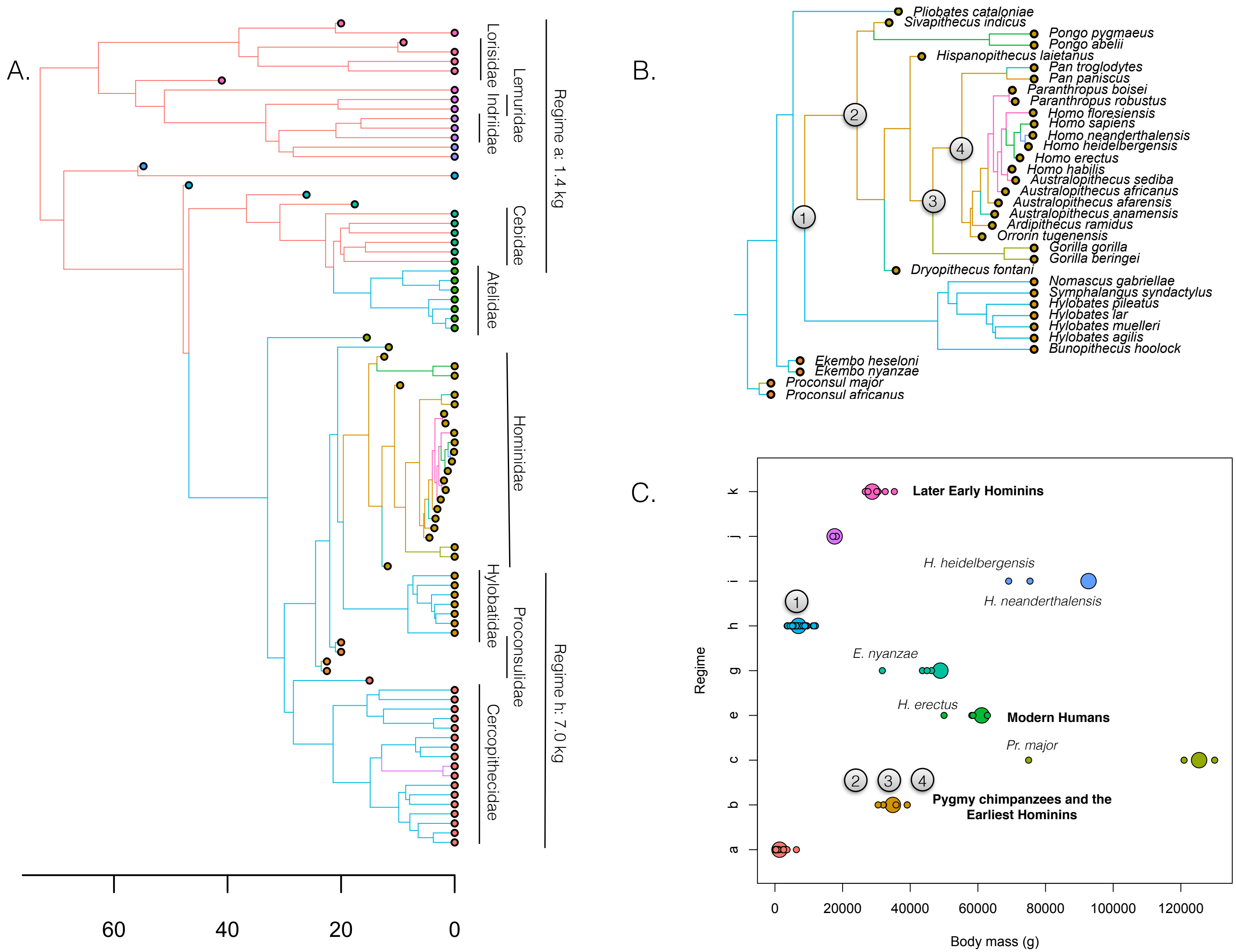

Supplementary Figure 2: Same as Figure 1 but using with smaller estimates of *Ardipithecus* and *Orrorin*.

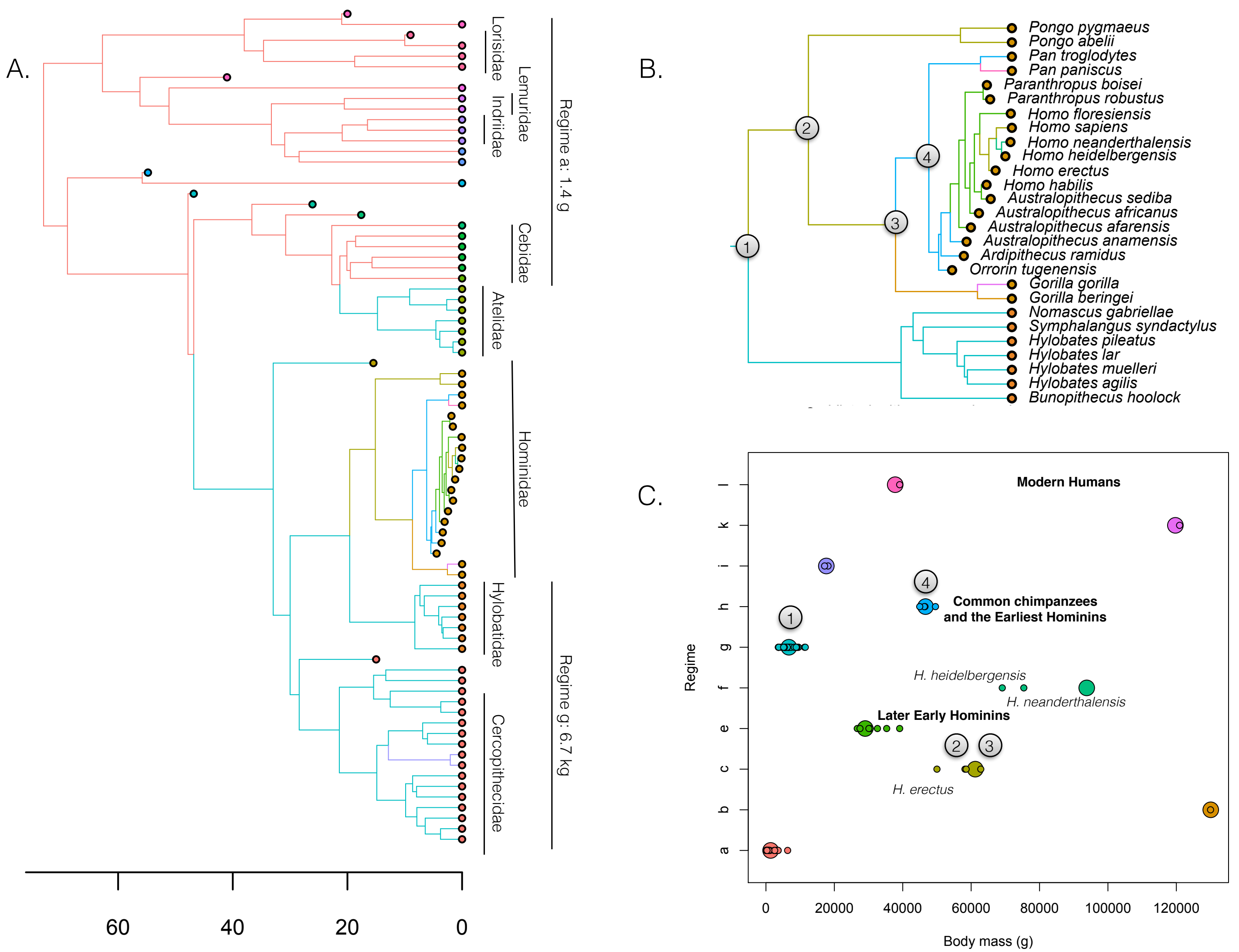

Supplementary Figure 3: Same as Figure 1 but without Miocene fossil apes.

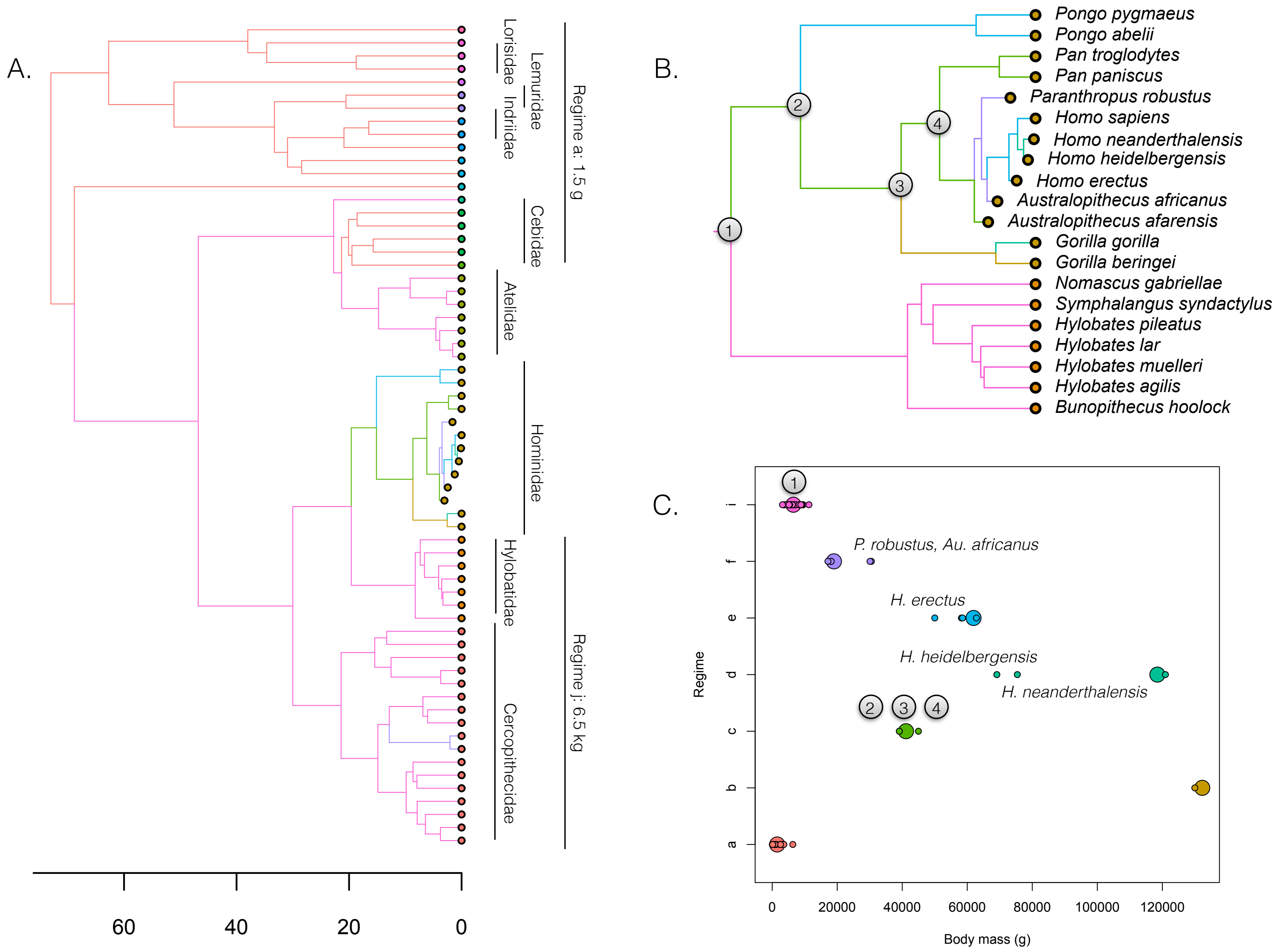

Supplementary Figure 4: Same as Figure 1 but with only well-sampled undisputed early hominins without other fossil primates.

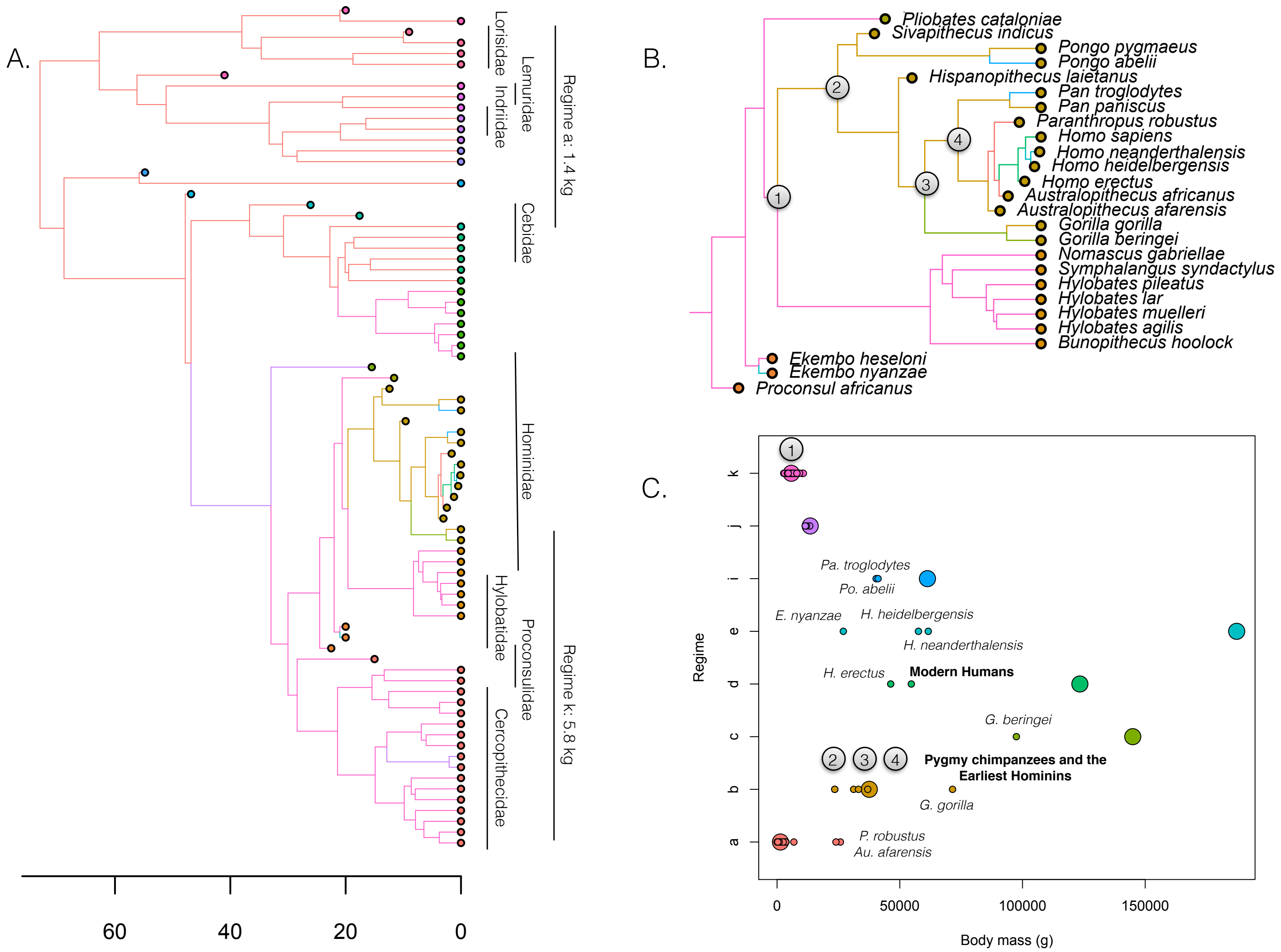

Supplementary Figure 5: Same as Figure 1 but with only female averages including well-sampled reliably-attributed early hominins.

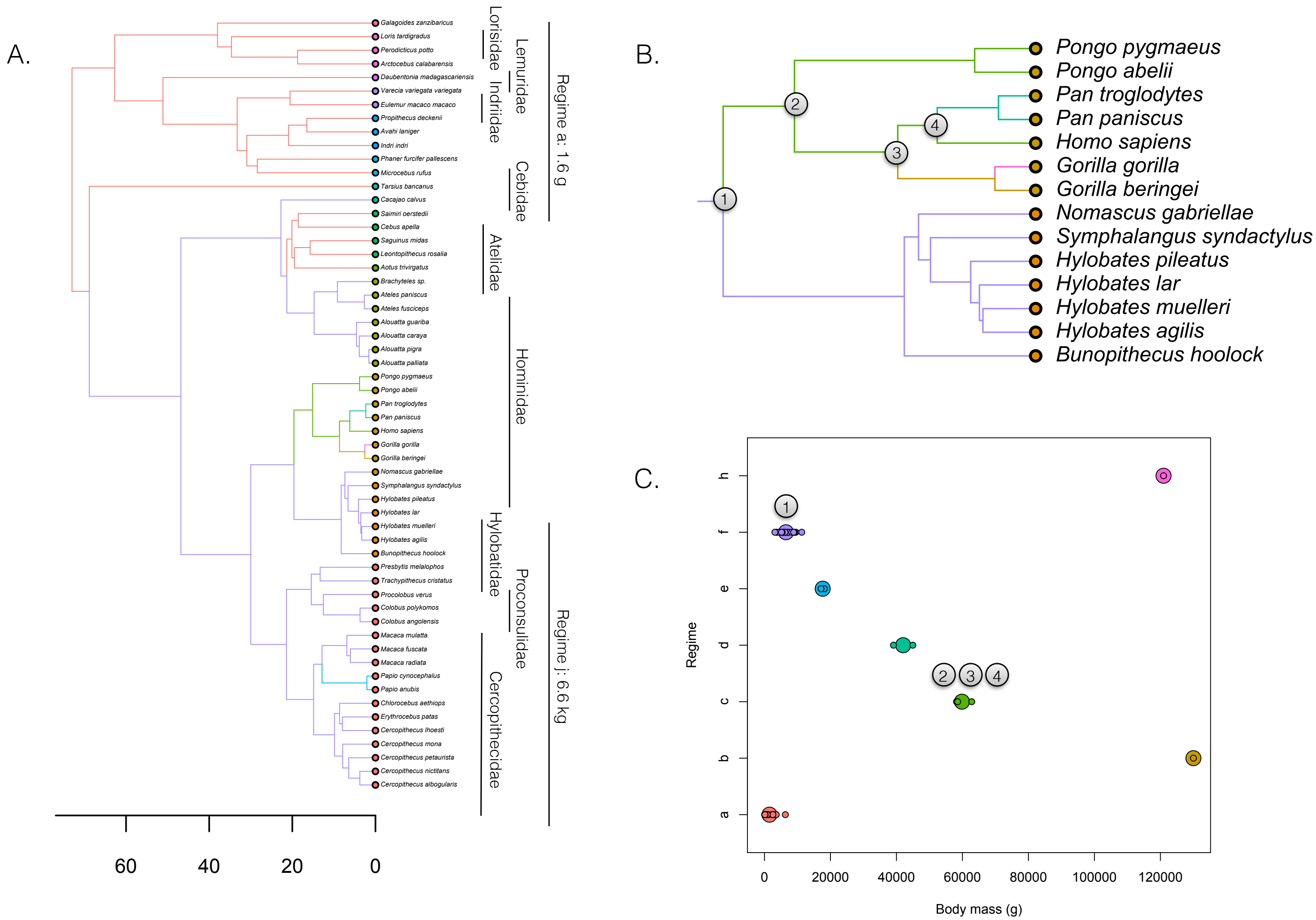

Supplementary Figure 6: Same as Figure 1 but with only extant primates.

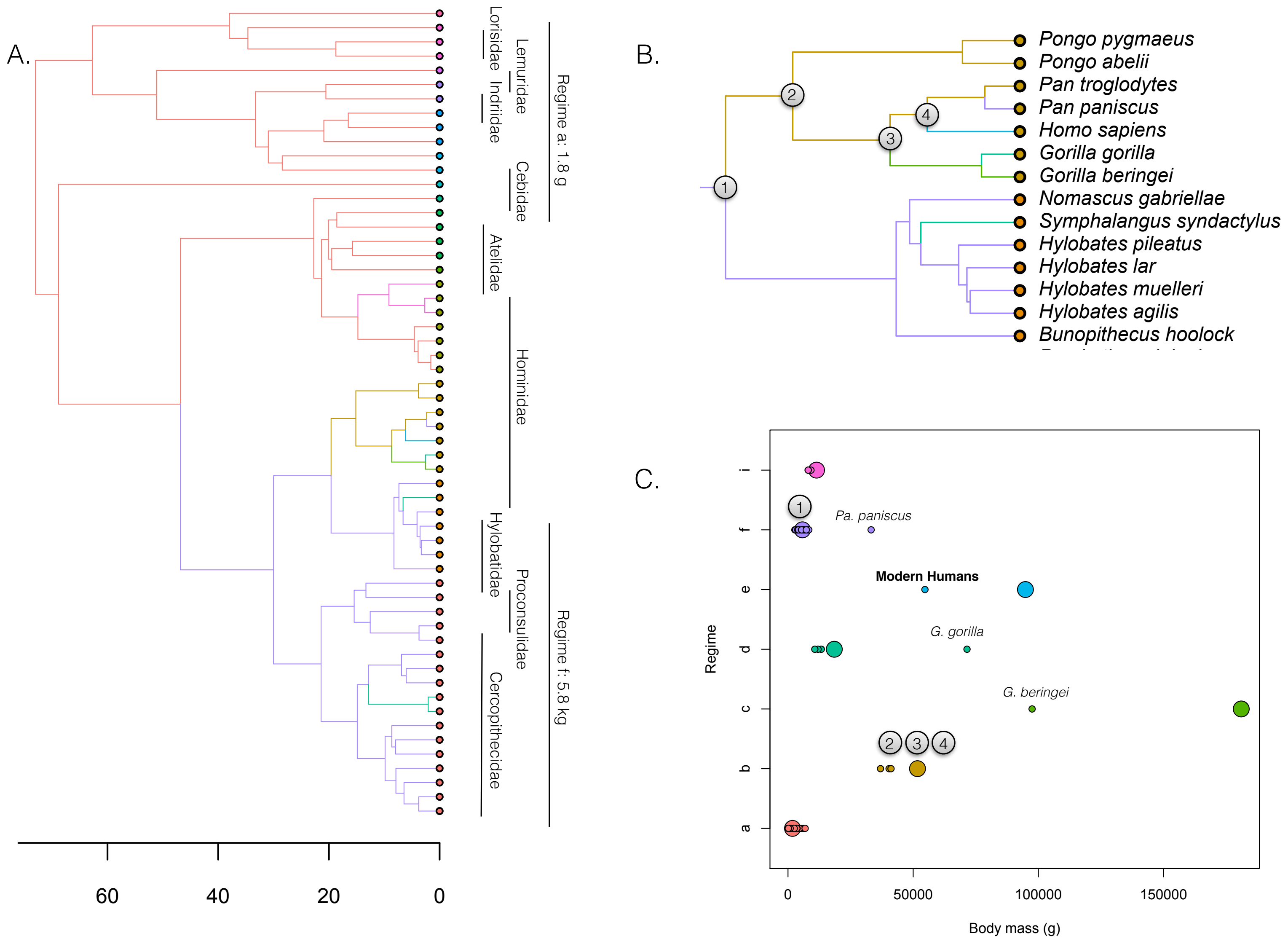

Supplementary Figure 7: Same as Figure 1 but with only extant female primates.

Supplementary References

1. Grabowski, M., Hatala, K. G., Jungers, W. L. & Richmond, B. G. Body mass estimates of hominin fossils and the evolution of human body size. *J. Hum. Evol.* **85,** 75–93 (2015).
2. Almécija, S., Smaers, J. B. & Jungers, W. L. The evolution of human and ape hand proportions. *Nat. Commun.* **6,** 7717 (2015).
3. Arsuaga, J. L. *et al.* Postcranial morphology of the middle Pleistocene humans from Sima de los Huesos, Spain. *Proc. Natl. Acad. Sci.* **112**, 11524-11529 (2015).
4. Ruff, C. B., Trinkaus, E. & Holliday, T. W. Body mass and encephalization in Pleistocene *Homo*. *Nature* **387,** 173–176 (1997).
5. Harrison, T. in *Cenozoic Mammals of Africa* (eds. Werdelin, L. & Sanders, W. J.) 429–469 (University of California Press, 2010).
6. Moyà-Solà, S. *et al.* First partial face and upper dentition of the Middle Miocene hominoid i from Abocador de Can Mata (Vallès-Penedès Basin, Catalonia, NE Spain): taxonomic and phylogenetic implications. *Am. J. Phys. Anthropol.* **139,** 126–145 (2009).
7. Alba, D. M., Almécija, S., Casanovas-Vilar, I., Méndez, J. M. & Moyà-Solà, S. A partial skeleton of the fossil great ape *Hispanopithecus laietanus* from Can Feu and the mosaic evolution of crown-hominoid positional behaviors. **7,** e39617 (2012).
8. Morgan, M. E. *et al.* A partial hominoid innominate from the Miocene of Pakistan: description and preliminary analyses. *Proc. Natl. Acad. Sci.* **112,** 82–87 (2015).
9. Alba, D. M. *et al.* Miocene small-bodied ape from Eurasia sheds light on hominoid evolution. *Science* **350,** aab2625–aab2625 (2015).
10. Steiper, M. E. & Seiffert, E. R. Evidence for a convergent slowdown in primate molecular rates and its implications for the timing of early primate evolution. *Proc. Natl. Acad. Sci.* **109,** 6006–6011 (2012).
11. Seiffert, E. R., Simons, E. L. & Attia, Y. Fossil evidence for an ancient divergence of lorises and galagos. *Nature* **422,** 421–424 (2003).
12. Harrison, T. New postcranial remains of *Victoriapithecus* from the middle Miocene of Kenya. *J. Hum. Evol.* **18,** 3–54 (1989).
13. Ni, X. *et al.* The oldest known primate skeleton and early haplorhine evolution. *Nature* **498,** 60–64 (2013).
14. Egi, N., Takai, M., Shigehara, N. & Tsubamoto, T. Body Mass Estimates for Eocene Eosimiid and Amphipithecid Primates Using Prosimian and Anthropoid Scaling Models. *International Journal of Primatology* **25,** 211–236 (2004).
15. Smith, R. J. & Jungers, W. L. Body mass in comparative primatology. *J. Hum. Evol.* **32,** 523–559 (1997).
16. Almécija, S. *et al.* The femur of *Orrorin tugenensis* exhibits morphometric affinities with both Miocene apes and later hominins. *Nat. Commun.* **4,** 2888 (2013).
17. White, T. D. *et al. Ardipithecus ramidus* and the Paleobiology of Early Hominids. *Science* **326,** 64 (2009).
18. Leakey, M. G., Feibel, C. S., McDougall, I., Ward, C. & Walker, A. New specimens and confirmation of an early age for *Australopithecus anamensis*. *Nature* **393,** 62–66 (1998).
19. Leakey, R. E. New hominid fossils from the Koobi Fora formation in northern Kenya. *Nature* **261,** 574–576 (1976).
20. Herries, A. I. R. & Adams, J. W. Clarifying the context, dating and age range of the Gondolin hominins and *Paranthropus* in South Africa. *J. Hum. Evol.* **65,** 676–681 (2013).
21. Pickering, R. *et al. Australopithecus sediba* at 1.977 Ma and implications for the origins of

the genus *Homo*. *Science* **333,** 1421–1423 (2011).
22. Feibel, C. S., Brown, F. H. & McDougall, I. Stratigraphic context of fossil hominids from the Omo Group deposits: northern Turkana Basin, Kenya and Ethiopia. *Am. J. Phys. Anthropol.* **78,** 595–622 (1989).
23. Herries, A. I. R., Curnoe, D. & Adams, J. W. A multi-disciplinary seriation of early *Homo* and *Paranthropus* bearing palaeocaves in southern Africa. *Quaternary International* **202,** 14–28 (2009).
24. Kimbel, W. H., Johanson, D. C. & Rak, Y. Systematic assessment of a maxilla of *Homo* from Hadar, Ethiopia. **103,** 235–262 (1997).
25. Kimbel, W. H. & Villmoare, B. From *Australopithecus* to *Homo*: the transition that wasn't. *Philos Trans R Soc Lond B Biol Sci* **371,** 20150248–10 (2016).
26. Sutikna, T. *et al.* Revised stratigraphy and chronology for *Homo floresiensis* at Liang Bua in Indonesia. *Nature* **532,** 366–369 (2016).
27. Wagner, G. A., Maul, L. C., Löscher, M. & Schreiber, H. D. Mauer - the type site of *Homo heidelbergensis*: palaeoenvironment and age. *Quaternary Science Reviews* **30,** 1464–1473 (2011).
28. Rink, W. J., Schwarcz, H. P., Smith, F. H. & Radovĉić. ESR ages for Krapina hominids. *Nature* **378,** 24–24 (1995).
29. McDougall, I., Brown, F. H. & Fleagle, J. G. Stratigraphic placement and age of modern humans from Kibish, Ethiopia. *Nature Publishing Group* **433,** 733–736 (2005).
30. McNulty, K. P., Begun, D. R., Kelley, J., Manthi, F. K. & Mbua, E. N. A systematic revision of *Proconsul* with the description of a new genus of early Miocene hominoid. *J. Hum. Evol.* **84,** 42–61 (2015).
31. Begun, D. R. in *The Primate Fossil Record* (ed. Hartwig, W. C.) 221–240 (2002).
32. Fleagle, J. G. *Primate Adaptation and Evolution*. (Academic Press, 2013).
33. Takai, M., Anaya, F., Shigehara, N. & Setoguchi, T. New fossil materials of the earliest new world monkey, *Branisella boliviana*, and the problem of platyrrhine origins. *Am. J. Phys. Anthropol.* **111,** 263–281 (2000).
34. Gonzales, L. A., Benefit, B. R., McCrossin, M. L. & Spoor, F. Cerebral complexity preceded enlarged brain size and reduced olfactory bulbs in Old World monkeys. *Nat. Commun.* **6,** 1–9 (2015).
35. Beard, K. C. & Wang, J. The eosimiid primates (Anthropoidea) of the Heti Formation, Yuanqu Basin, Shanxi and Henan Provinces, People's Republic of China. *J. Hum. Evol.* **46,** 401–432 (2004).
36. Arnold, C., Matthews, L. J. & Nunn, C. L. The 10kTrees website: A new online resource for primate phylogeny. *Evol. Anthropol.* **19,** 114–118 (2010).
