# Supplementary material for: Evidence of a chimpanzee-sized ancestor of humans but a gibbon-sized ancestor of apes
Source: Nat Commun. 2017 Oct 12;8:880. doi: 10.1038/s41467-017-00997-4 (PMC5638852; doi:10.1038/s41467-017-00997-4)
Supplement: Supplementary file 2 — Description of Additional Supplementary Files [file 41467_2017_997_MOESM2_ESM.pdf]

File Name: Supplementary Data 1
Description: Phylogenetic tree used in main analysis with branch lengths.
