# Supplementary material for: Evidence of a chimpanzee-sized ancestor of humans but a gibbon-sized ancestor of apes
Source: Nat Commun. 2017 Oct 12;8:880. doi: 10.1038/s41467-017-00997-4 (PMC5638852; doi:10.1038/s41467-017-00997-4)
Supplement: Supplementary file 3 — Supplementary Data [file 41467_2017_997_MOESM3_ESM.pdf]

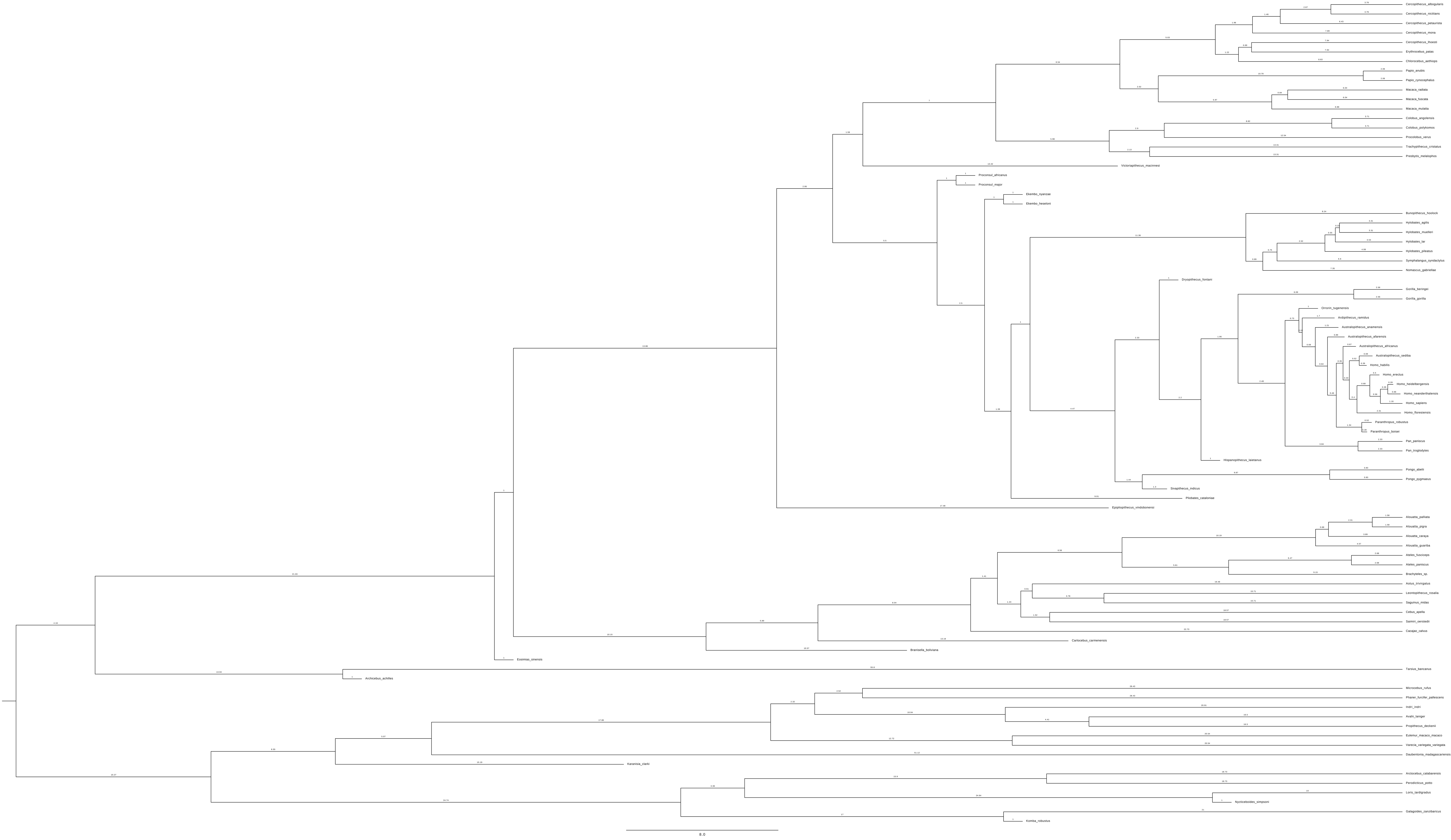
Cercopithecus_albogularis
Cercopithecus_nictitans
Cercopithecus_petaurista
Cercopithecus_mona
Cercopithecus_lhoesti
Erythrocebus_patas
Chlorocebus_aethiops
Papio_anubis
Papio_cynocephalus
Macaca_radiata
Macaca_fuscata
Macaca_mulatta
Colobus_angolensis
Colobus_polykomos
Procolobus_verus
Trachypithecus_cristatus
Presbytis_melalophos
Victoriapithecus_macinnesi
Proconsul_africanus
Proconsul_major
Ekembo_nyanzae
Ekembo_heseloni
Bunopithecus_hoolock
Hylobates_agilis
Hylobates_muelleri
Hylobates_lar
Hylobates_pileatus
Symphalangus_syndactylus
Nomascus_gabriellae
Dryopithecus_fontani
Gorilla_beringei
Gorilla_gorilla
Orrorin_tugenensis
Ardipithecus_ramidus
Australopithecus_anamensis
Australopithecus_afarensis
Australopithecus_africanus
Australopithecus_sediba
Homo_habilis
Homo_erectus
Homo_heidelbergensis
Homo_neanderthalensis
Homo_sapiens
Homo_floresiensis
Paranthropus_robustus
Paranthropus_boisei
Pan_paniscus
Pan_troglodytes
Hispanopithecus_laietanus
Pongo_abelii
Pongo_pygmaeus
Sivapithecus_indicus
Pliobates_cataloniae
Epipliopithecus_vindobonensi
Alouatta_palliata
Alouatta_pigra
Alouatta_caraya
Alouatta_guariba
Ateles_fusciceps
Ateles_paniscus
Brachyteles_sp.
Aotus_trivirgatus
Leontopithecus_rosalia
Saguinus_midas
Cebus_apella
Saimiri_oerstedii
Cacajao_calvus
Carlocebus_carmenensis
Branisella_boliviana
Eosimias_sinensis
Tarsius_bancanus
Archicebus_achilles
Microcebus_rufus
Phaner_furcifer_pallescens
Indri_indri
Avahi_laniger
Propithecus_deckenii
Eulemur_macaco_macaco
Varecia_variegata_variegata
Daubentonia_madagascariensis
Karanisia_clarki
Arctocebus_calabarensis
Perodicticus_potto
Loris_tardigradus
Nycticeboides_simpsoni
Galagoides_zanzibaricus
Komba_robustus
8.0
